# Retraction Note: Pancreatic cancer-initiating cell exosome message transfer into noncancer-initiating cells: the importance of CD44v6 in reprogramming

**DOI:** 10.1186/s13046-025-03339-1

**Published:** 2025-02-28

**Authors:** Zhe Wang, Hanxue Sun, Jan Provaznik, Thilo Hackert, Margot Zöller

**Affiliations:** 1 Department of Oncology, The First Affiliated Hospital of Guangdong Pharmaceutical University, Guangzhou, China; 2 Pancreas Section, University Hospital of Surgery, Im Neuenheimer Feld 110, D69120 Heidelberg, Germany; 3 Gene Core Unit, EMBL, Heidelberg, Germany

**Retraction Note:** ***J Exp Clin Cancer Res*** **38, 132 (2019)**

10.1186/s13046-019-1129-8

The Editor-in-Chief has retracted this article. A number of image duplications were brought to the attention of the publisher by a third party. While the manuscript was under investigation, the authors requested a correction, which was published. The raw data were not supplied by the author upon request for verification. The Editor and Publisher have lost confidence in the integrity of the data in this article.

Author Zhe Wang disagrees with this retraction. All other authors have not responded to correspondence from the publisher regarding this retraction.

